# Comparison of Rhythmic Auditory Stimulation Gait Training with and Without Vibrotactile Feedback on Balance and Gait in Persons with Stroke: A Randomized Controlled Trial

**DOI:** 10.3390/bioengineering12111177

**Published:** 2025-10-29

**Authors:** Su-Jin Kim, Sun-Min Kim, Sang-Hun Jang

**Affiliations:** 1 Department of Health and Medical Sciences, Graduate School of Physical Therapy, Korea National University of Transportation, Jeungpyeong-gun 27909, Chungcheongbuk-do, Republic of Korea; sj960208@naver.com; 2 Department of Physical Therapy, Gimcheon University, Gimcheon-si 39528, Gyeongsangbuk-do, Republic of Korea; jjssppaarrkk@hanmail.net; 3 Department of Physical Therapy, Korea National University of Transportation, Jeungpyeong-gun 27909, Chungcheongbuk-do, Republic of Korea

**Keywords:** balance, gait, rhythmic auditory stimulation, stroke, vibrotactile feedback

## Abstract

Background: Although both rhythmic auditory stimulation (RAS) and vibrotactile feedback have been shown to yield beneficial effects in stroke rehabilitation, evidence regarding their combined application remains limited. This study investigates the effects of RAS gait training alone (RG) versus RAS combined with vibrotactile feedback (RAS-V) on balance and gait in individuals post-stroke. Methods: Twenty-two people with stroke were randomly assigned to either an RAS-V or an RG group. The RAS-V group performed RAS gait training combined with vibrotactile feedback while the RG group performed RAS gait training. Both groups participated in 30-min gait training sessions, 5 times a week for 4 weeks. Balance ability was assessed using the Berg Balance Scale (BBS) and Timed Up and Go test (TUG). Gait ability was evaluated using the G-Walk gait analyzer and the 10-m Walk Test (10 mWT), including gait cadence, velocity, and stride length. Results: Within-group comparisons showed significant improvements in BBS (*p* < 0.001) and TUG scores (*p* < 0.05) in both groups. The RAS-V group demonstrated significant post-intervention improvements in gait velocity, 10 mWT (*p* < 0.05), and gait cadence (*p* < 0.001). Between-group comparisons revealed that the RAS-V group achieved significantly greater improvements than the RG group in TUG, gait cadence, gait velocity, and 10 mWT (*p* < 0.05). Conclusions: RAS gait training with vibrotactile feedback enhances balance and gait ability more effectively than RAS gait training alone, suggesting additional benefits of incorporating vibrotactile feedback.

## 1. Introduction

Stroke, caused by cerebrovascular occlusion or hemorrhage, often leads to persistent neurological deficits lasting over 24 h [1], typically resulting in contralateral motor, sensory, perceptual, and language impairments that limit mobility, activities of daily living, and postural control, thereby contributing to balance and gait disturbances [2,3,4]. Balance deficits in individuals with stroke often arise from weakness and abnormal muscle activation on the paretic side, which lead to lateral asymmetry; such persistent asymmetrical movement patterns interfere with postural reflexes and equilibrium responses, thereby increasing the risk of falls [5,6,7]. Gait is a critical function for maintaining mobility in daily life, yet approximately 22% of individuals with stroke fail to regain independent ambulation [8,9]. Stroke-related gait characteristics, such as reduced gait speed, circumduction of the paretic limb, excessive knee extension, and gait asymmetry, further hinder compensatory reactions during balance loss, thereby increasing the risk of falls [10,11,12,13].

Recent studies have reported positive outcomes from biofeedback-based interventions for improving gait in individuals with stroke [14,15,16]. Biofeedback is a rehabilitation training method that enhances motor learning and performance by providing real-time visual or auditory information about the body’s neurophysiological signals through specialized devices [17]. Such external feedback helps individuals develop internal awareness of movement goals, and learning motor tasks with external feedback has been shown to improve retention of motor skills compared to training without feedback [18,19]. Various biofeedback modalities, including auditory, visual, tactile, and vibrotactile feedback, have been used to optimize motor learning. Among these, rhythmic auditory stimulation (RAS) is widely applied because regular rhythmic cues can synchronize motor and sensory regions, activate extensive brain networks, and improve motor function [20]. Its effectiveness is supported by strong neuroanatomical connectivity between auditory and motor areas and by the auditory system’s faster stimulus processing (20–50 ms) compared to visual or tactile inputs [21,22,23,24]. Similarly, haptic vibrotactile feedback has been reported to improve balance control and postural stability in individuals with stroke and vestibular disorders [25,26].

Since the 1990s, numerous studies have investigated these biofeedback modalities for neurological rehabilitation. For example, Thaut et al. (1997) [27] demonstrated that gait training with RAS increased stride length and walking speed in individuals with stroke, and Suh et al. (2014) [28] reported that progressive gait training with RAS improved balance and gait parameters more effectively than conventional training. Kil et al. (2021) [25] further reported that real-time vibrotactile feedback improved static balance and weight-bearing symmetry. These findings suggest that biofeedback-based training, when supervised by therapists, not only facilitates motor learning but also promotes patients’ sense of accomplishment and enhances their independence in gait practice [29].

However, the results of previous studies on RAS gait training alone in individuals with stroke have been inconsistent. While some studies have reported positive outcomes, the overall findings remain varied [30,31]. The effects of individual biofeedback interventions, such as gait training using RAS and treadmills [32,33] and vibrotactile feedback [26,34,35], have been demonstrated in previous studies. Auditory feedback facilitates synchronization of lower limb movements through rapid signal processing, while tactile feedback provides real-time input to support error correction during motor performance. During gait training, extrinsic feedback allows individuals to adjust their movements independently, thereby enhancing gait ability [36]. The combination of the two feedback modalities may enhance motor learning by facilitating movement correction during walking.

Despite this potential, there is a lack of well-designed intervention programs that combine both RAS and vibrotactile feedback. Research on the effectiveness of such combined training remains insufficient. Based on these findings, this study investigates whether integrating vibrotactile feedback with RAS gait training can provide additional sensory input to support attentional focus and motor control during walking. The goal is to determine whether this combined approach offers greater benefits for gait performance than using RAS alone. Accordingly, the present study hypothesized that the combined application of RAS and vibrotactile feedback during gait training would lead to improvements in both balance and gait performance.

Therefore, this study aimed to investigate the effects of RAS gait training (RG) combined with vibrotactile feedback (RAS-V) on balance and gait abilities in individuals with stroke.

## 2. Materials and Methods

### 2.1. Participants

This study recruited individuals with stroke who were receiving inpatient treatment at a rehabilitation hospital in Daejeon-si and met the eligibility criteria. The inclusion criteria were as follows: Participants in the subacute phase (1–6 months after stroke diagnosis), with a Mini-Mental State Examination-Korean (MMSE-K) score of ≥24, who were able to communicate effectively and could walk independently for at least 14 m, regardless of gait-aid use. Participants were also required to have a Functional Ambulation Category (FAC) score ≥ 3, indicating the ability to walk under supervision without physical assistance. Additionally, all participants were required to understand the purpose of the study and provide written informed consent.

The exclusion criteria were as follows: participants with unilateral neglect, spatiotemporal disorders, psychiatric disorders, other neurological conditions besides stroke, severe rheumatoid arthritis, osteoarthritis, a history of orthopedic surgery affecting the musculoskeletal system, sensory impairments such as hearing or visual disabilities, medical conditions (e.g., angina or chronic obstructive pulmonary disease) that could impact the cardiovascular or respiratory system, and cognitive impairments preventing them from understanding treatment instructions.

### 2.2. Research Procedure

This study was a randomized controlled trial designed to compare the effects between RAS-V and RG groups. The required sample size was calculated using the G*Power version 3.1 program (Heinrich Heine University, Düsseldorf, Germany). The allocation ratio was set at 1:1, with a significance level of 0.05 and a power of 0.8. Based on a previous study [37], an effect size of 1.0 was adopted, resulting in a required total of 28 participants. To account for potential dropout, 30 participants were recruited, with 15 assigned to the RAS-V group and 15 to the RG group. Participants were randomly assigned to the experimental or control group through a simple randomization method, where slips of paper labeled “O” (RAS-V group) and “X” (RG group) were drawn from a basket. The study employed a single-blind design, ensuring that participants were unaware of their group assignment. Allocation concealment, participant enrollment, and group assignment were conducted by the author.

**Figure 1:**
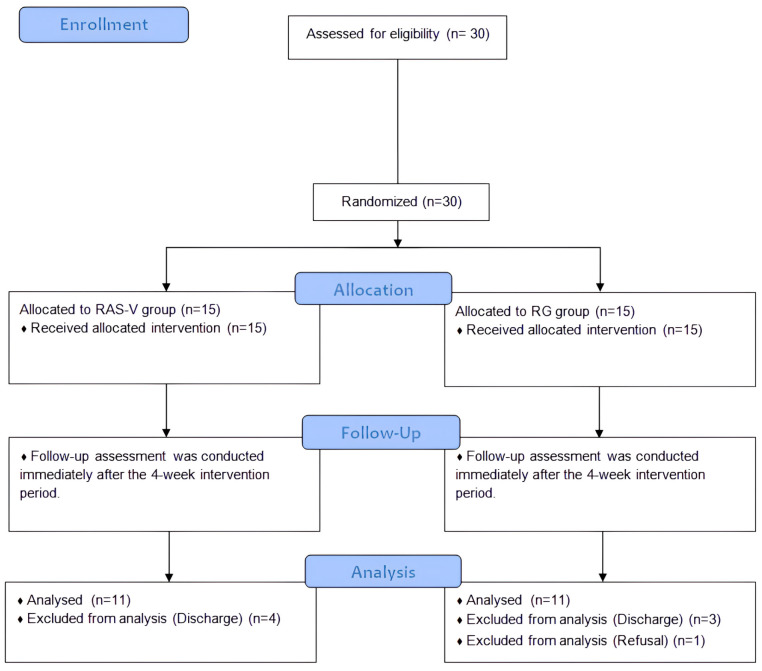
Flow diagram. Abbreviations: RAS-V, Rhythmic auditory stimulation gait training combined with vibrotactile feedback; RG, Rhythmic auditory stimulation gait training.

The study period was from 30 September to 4 November 2024. Pre-intervention assessments were conducted by a physical therapist with more than 5 years of clinical experience and independent of the study, and the assessor was blinded to group allocation. The intervention was systematically administered once daily for 30 min, 5 times per week for 4 weeks. Post-intervention assessments were conducted using the same measurement tools by the same assessor who had performed the pre-intervention assessments. This assessor was not involved in the intervention and remained blinded to the participants’ group assignments throughout the study. Participants were informed about the intervention methods specific to each group, the intervention duration per session, the total study period, the assessment tools used pre- and post-intervention, and the provision of rest periods as needed. A total of 30 participants were enrolled prior to randomization; however, before the final assessment, one declined participation and seven were discharged during the training sessions, resulting in 22 participants who completed the post-intervention assessments (Figure 1). The study was conducted in accordance with the Declaration of Helsinki and received approval from the Institutional Review Board of Korea National University of Transportation (Approval number: KNUT-2024-HR-16-29).

### 2.3. Intervention

**Figure 2:**
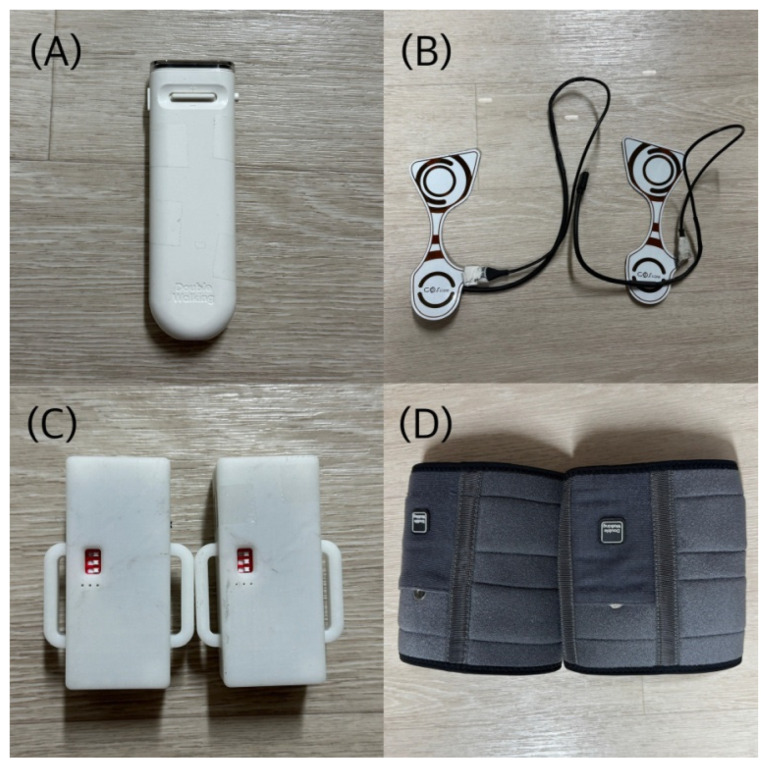
Intervention tools: (**A**) Handheld device (Auditory stimulus feedback module, GOS, Gyeongsan-si, Republic of Korea); (**B**) pad-type foot pressure sensor (Gait measurement module, GOS, Gyeongsan-si, Republic of Korea); (**C**) transmitter; (**D**) calf brace.

The equipment used in this study included a handheld device (Auditory stimulus feedback module, GOS, Ltd., Gyeongsan-si, Republic of Korea) that provided RAS for gait, a pad-type pressure sensor (Gait measurement module, GOS, Ltd., Gyeongsan-si, Republic of Korea) that measured the starting points of stance phases and swing phases during gait, a transmitter, and a calf brace (Figure 2). The auditory stimulus feedback and gait measurement modules were custom-built research devices manufactured by GOS. The handheld device consists of a setting section to adjust the rhythm of auditory stimuli at intervals, an auditory stimulus provider, a vibration unit for feedback delivery, and a communication section that interacts with the pressure sensor. The device was operated with a power on–off switch and two control buttons: pressing the left button activated vibrotactile feedback, while the central button was moved upward (+) to increase or downward (−) to decrease the RAS tempo. The auditory stimulus was delivered at an initial interval of 0.8 s (800 ms) and could be adjusted in 50 ms increments across 20 levels, allowing stepwise modulation of the stimulus interval within a range of 0.8–1.8 s according to the participant’s gait pattern. The interval could then be adjusted in increments of 50 ms, which corresponds to approximately 6.25% change relative to the initial interval. This stepwise adjustment allowed modulation of the interval within a range of 0.8–1.8 s (up to 125% increase relative to baseline) according to each participant’s gait characteristics and tolerance. Vibrotactile feedback was provided via a miniature vibration motor (3 V, 60 mA, 13,000 RPM) that was activated for 0.1 s whenever the difference between the auditory cue and the foot-contact timing exceeded 250 ms. The vibrotactile feedback was delivered at a frequency of 200–230 Hz and an amplitude of 0.5–1.2 mm, which is sufficient for participants to perceive through the skin.

Before each intervention session, participants wore calf braces, attached the transmitter, and positioned the pad-type pressure sensors on the insoles of both shoes, which were connected to the transmitter via wired communication. Participants held the handheld device with their non-affected hand and performed gait training synchronized to the periodically delivered RAS generated by the device. Data from the foot pressure sensors were transmitted to the handheld device in real time. When the difference between the RAS signal and the timing of foot contact exceeded 250 ms, vibrotactile feedback was immediately triggered through the handheld device. This provision of vibrotactile feedback to the hand helped guide participants to adjust their steps to align with the auditory cues, promoting a consistent gait pattern.

Each gait training session lasted 30 min, including 3 min of warm-up, 25 min of main exercise, and 2 min of cool-down. During the 25-min main training, participants in both groups repeatedly walked on an indoor level-ground walkway at their comfortable walking speed using the same equipment. In this study, the comfortable walking speed was defined as the speed at which each participant walked on an indoor level-ground walkway following the instruction, “Please walk comfortably.” The RAS-V group performed gait training synchronized to the RAS interval while also receiving real-time vibrotactile feedback. To encourage motor learning, participants in the RAS-V group were instructed to minimize the triggering of vibrotactile feedback by voluntarily adjusting their steps to the auditory rhythm. The RG group performed gait training using RAS alone, without vibrotactile feedback. The key difference between the two groups was the presence or absence of vibrotactile feedback. The interval of the RAS was initially set to match each participant’s comfortable gait cadence; as participants adapted, the interval was gradually adjusted in a stepwise manner according to individual tolerance to increase the training difficulty. For example, longer intervals were applied for participants with faster walking speeds, and shorter intervals were applied for those with slower walking speeds to modulate task difficulty. If a participant was unable to adapt to the adjusted interval, no further increase in difficulty was attempted. All training sessions were conducted under one-on-one supervision by a therapist to ensure safety and to provide assistance as needed. Rest periods were provided as needed at the participant’s request. In addition to the experimental intervention, both groups received an additional 30 min of conventional physical therapy, consisting of neurodevelopmental treatment (NDT) aimed at improving balance and gait abilities. The gait training protocol was adapted from previously published methods, and a summary of the intervention program is provided in Table 1 [38].

### 2.4. Evaluation

To assess changes in balance and gait within and between the groups, all participants underwent pre- and post-intervention assessments. Balance ability was evaluated using the Berg Balance Scale (BBS) and the Timed Up and Go (TUG) test, while gait ability was assessed using the G-Walk system (G-Walk, BTS Bioengineering, Milan, Italy) and the 10-Meter Walk Test (10 mWT).

The BBS, a standardized 14-item scale scored from 0 to 4, is widely used to assess balance and fall risk, with higher scores indicating better balance. The TUG is a simple and widely used test that measures mobility and fall risk [39,40] by timing participants as they stand up from a chair, walk 3 m, turning, returning, and sitting down.

Gait parameters, including gait cadence, velocity, and stride length, were recorded using a waist-worn sensor of the G-Walk system. The 10 mWT, a validated test for assessing walking speed, was conducted along a 14-m straight path, timing only the central 10 m to exclude acceleration and deceleration. The test was repeated three times, and the average time was recorded; the use of walking aids was documented.

### 2.5. Analysis

All data analyses in this study were conducted using SPSS version 24.0 (IBM Corp., Armonk, NY, USA). The normality of the data was tested using the Shapiro–Wilk test. To assess the homogeneity of participants’ general characteristics, chi-squared tests and independent *t*-tests were performed. To examine within-group changes in balance and gait before and after the intervention, paired *t*-tests were conducted for both the RAS-V and RG groups. For between-group differences in changes before and after the intervention, independent *t*-tests were used. Additionally, effect sizes were calculated using Cohen’s d, and 95% confidence intervals were analyzed. A significance level of *p* < 0.05 was set for all statistical analyses.

## 3. Results

### 3.1. General Characteristics of the Participants

This study initially enrolled 30 participants, but after excluding 8 individuals due to withdrawal and discharge, data from 22 participants were analyzed. There were no statistically significant differences between the two groups in gender, age, weight, height, lesion type, lesion side, and MMSE-K scores. This confirmed the homogeneity of the groups in terms of their general characteristics. The general characteristics of the participants are presented in Table 2.

### 3.2. Pretreatment Comparisons of Dependent Variables

No significant differences were found between the RAS-V and RG groups in the dependent variables, including the BBS, TUG, gait cadence, gait velocity, stride length, and 10 mWT. These results confirm the homogeneity of the dependent variables between the two groups prior to the intervention. The results of the pre-homogeneity test are presented in Table 3.

### 3.3. Change in Balance

Both the RAS-V and RG groups showed significant improvements in the average BBS scores from pre- to post-intervention (*p* < 0.001). However, when comparing the mean change in BBS scores between the groups, there was no statistically significant difference (*p* = 0.29).

**Figure 3:**
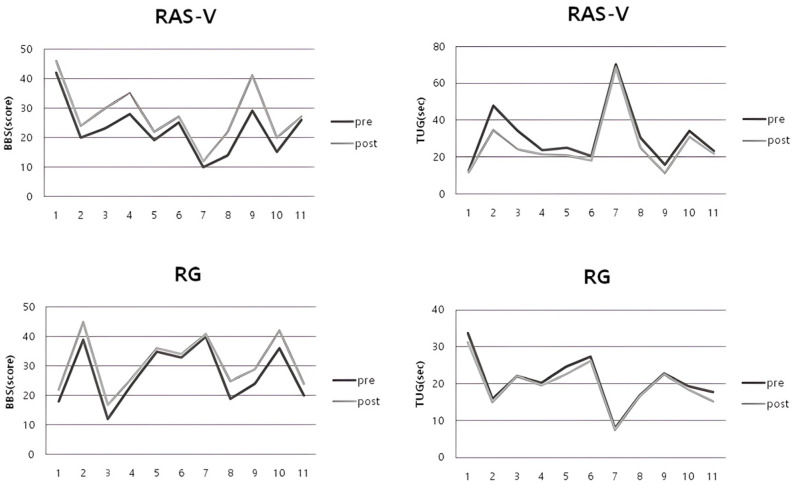
Individual pre–post changes in BBS and TUG for each group.

For the TUG, the RAS-V group and RG group’s average times showed a significant improvement at post-intervention (RAS-V group: *p* = 0.003; RG group: *p* = 0.004). When comparing the mean change in TUG times between the groups, there was a statistically significant difference (*p* = 0.013). Notably, the TUG times of participants S2 and S3 in the RAS-V group decreased by approximately 27.7% and 28.7%, respectively, at post-intervention. Table 4 shows the changes in balance ability for both groups. Figure 3 shows the pre- and post-intervention changes for individual participants in each group.

### 3.4. Change in Gait

**Figure 4:**
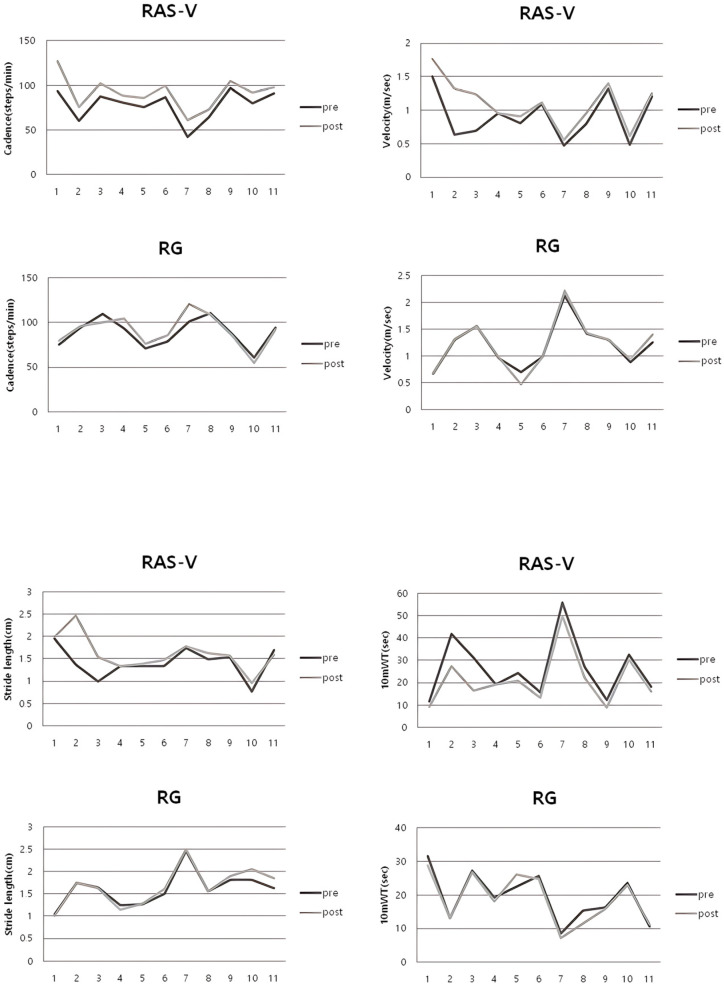
Individual pre–post changes in gait cadence, velocity, stride length, and 10 mWT for each group.

The RAS-V group showed significant post-intervention improvements in gait cadence (*p* < 0.001), gait velocity (*p* = 0.017), and 10 mWT (*p* = 0.006), whereas the RG group showed no significant changes in these measures (cadence: *p* = 0.31; velocity: *p* = 0.65; 10 mWT: *p* = 0.28). There was a statistically significant difference between the groups in the mean changes in gait ability measures—gait cadence (*p* = 0.004), gait velocity (*p* = 0.028), and 10 mWT (*p* = 0.015). For stride length, there was no significant difference in within-group and between-group comparisons. The gait velocity of participants S2 and S3 in the RAS-V group increased by approximately 106.3% and 79.7%, respectively, at post-intervention. Table 5 shows the changes in gait ability for both groups. Figure 4 shows the pre- and post-intervention changes for individual participants in each group.

## 4. Discussion

This study aimed to compare the effects of RAS gait training, with and without vibrotactile feedback, on balance and gait in persons with stroke. Both groups showed significant improvements in balance, as reflected by higher BBS scores and shorter TUG times. The RAS-V group achieved a significantly greater reduction in TUG time compared to the RG group.

RAS has long been employed as an intervention to enhance balance in individuals with stroke and was applied to both groups in the present study. In the systematic review and meta-analysis by Wang et al. (2022) [41], RAS was found to improve key gait parameters, such as walking velocity, step length, and cadence, as well as balance indicators including the BBS and the Overall Balance Index (OBI). The authors emphasized a lack of high-quality studies focusing specifically on balance ability, indicating the need for further research. Therefore, the present study aimed to investigate the effects of RAS gait training combined with vibrotactile feedback on balance and gait improvement. Xu et al. (2017) [42] developed a wearable vibrotactile feedback system and, when applied to healthy older adults, it reduced trunk tilt, increased balance maintenance time, and enabled the adoption of a new gait pattern within two minutes. Although conducted in healthy older adults, this study highlights the capacity of vibrotactile feedback to rapidly modify movement strategies, supporting its potential use in neurorehabilitation for individuals with stroke.

Both groups in the present study showed improvements in balance from pre- to post-intervention; however, no significant between-group differences were observed in BBS scores. Stevenson (2001) [43] reported that the minimum detectable change (MDC) of the BBS in patients with stroke is approximately 6 points at the 90% confidence level. This suggests that the lack of significant between-group differences observed in this study may be partly attributed to the limited sensitivity of the BBS in detecting clinically meaningful changes in balance. In addition, the reduced statistical power may have hindered the detection of a definitive intervention effect. While vibrotactile feedback did not yield greater benefits than RAS alone, these findings suggest that vibrotactile feedback may complement RAS by facilitating proprioceptive guidance during the stance phase, thereby supporting active motor control. Taylor et al. (2006) [44] reported that, when the 10-m walk test was performed in stroke patients in clinical settings, those with a gait speed of less than 0.8 m/s showed a significant difference between gait speeds measured in clinical settings and those measured in community environments. Therefore, gait speed significantly affects activities of daily living, especially mobility and walking performance. However, individuals with stroke typically walk slowly on the affected side than on the unaffected side, and their stride length on the affected side is often shorter [45]. Considering these gait characteristics, biofeedback has been widely utilized as a rehabilitation strategy for improving gait in individuals with stroke. Biofeedback enhances motor performance by employing various tools, such as visual, auditory, and tactile feedback [17].

With respect to gait performance, the RAS-V group showed significant pre- to post-intervention improvements in gait cadence, gait velocity, and the 10 mWT. Previous studies have reported similar benefits of vibrotactile feedback. Ma et al. (2018) [35] reported that providing real-time vibrotactile feedback to patients with stroke significantly reduced foot inversion and increased the plantar contact area of the paretic limb during mid-stance. Afzal et al. (2015) [34] and Kim et al. (2022) [46] reported that vibrotactile feedback can increase stance time and improve gait symmetry in individuals with stroke during gait training. De Angelis et al. (2021) [47] reported in their systematic review that vibrotactile feedback significantly improved balance control, gait speed, stride length, and walking stability; and also had a positive impact on activities of daily living. In line with the concept of knowledge of performance [19], vibrotactile feedback in this study provided direct information about cadence errors, likely supporting error detection and correction. When integrated with RAS, vibrotactile feedback may enhance motor control by improving foot-placement accuracy in response to auditory cues during dynamic gait tasks, thereby contributing to the greater improvements observed in the RAS-V group.

In consideration of the distinction between performance and learning emphasized in motor learning research, the results were interpreted not only on within-group improvements but also post-intervention between-group comparisons. Although no significant between-group differences were found in balance measures, the combination of vibrotactile feedback and RAS may have supported gait symmetry and dynamic stability during task performance. When considering its application to patients who have difficulty walking without assistance, we propose that combining vibrotactile feedback and RAS. This combined approach, supplemented by therapist support such as verbal cueing for rhythm synchronization or manual assistance with foot placement, may help reduce gait dependency and enhance gait stability. Therefore, the integration of auditory and vibrotactile feedback demonstrated in this study is meaningful in that encourage participants to engage more actively in modifying their gait patterns.

However, this study has several limitations. First, the number of participants who completed the study was smaller than the initially planned sample size, which limits the generalizability of the intervention effects. Second, it was difficult to isolate the effects of the intervention alone, as personal exercise activities and conventional physical therapy outside of the intervention program were not controlled. Third, the short intervention period prevented the evaluation of the long-term effects of combining vibrotactile feedback with RAS. Fourth, although NDT was conducted separately from the intervention program to avoid simultaneous performance, participant fatigue may still have influenced the outcome measures. Fifth, this study was retrospectively registered after participant enrollment and the completion of the intervention, which could affect the study’s methodological rigor. Sixth, the duration since stroke onset was not collected in detail, which limited our ability to assess its potential influence on individual recovery outcomes. Finally, participants were required to hold the device during gait training, which may limit its practicality in daily life. Despite these limitations, this study demonstrated that combining vibrotactile feedback with RAS may facilitate motor control during gait and improve gait function. However, further research is needed to establish this combined approach as a standardized intervention program.

Interestingly, two participants in the RAS-V group (S2 and S3) exhibited markedly greater improvements in gait velocity and TUG performance compared to other participants. This observation suggests potential inter-individual variability in responsiveness to the RAS-V training. In particular, S2 and S3 may represent strong responders to the intervention, showing pronounced adaptation to rhythmic and vibrotactile cues. Future studies with larger sample sizes are warranted to identify the characteristics and contributing factors associated with such enhanced responsiveness to RAS-V training.

Michelini et al. (2022) [48] reported that both the vibrotactile feedback group and the RAS group showed improvements in cadence and stance time symmetry compared to walking without feedback under gait asymmetry conditions, whereas no significant differences were observed in walking speed, stride length, or step length symmetry. However, vibrotactile feedback demonstrated a comparable effect to RAS in improving asymmetry, suggesting that future research should explore combining vibrotactile feedback with RAS to develop intervention programs that extend beyond conventional therapies. It is necessary to develop a comprehensive intervention program and further validate the findings of this study through randomized controlled trials with larger sample sizes. Considering the proportional recovery rule, which suggests that recovery potential may vary depending on the time since stroke onset, larger randomized controlled trials are needed to further validate these effects and to rigorously control for post-stroke duration. The observed benefits of combining multisensory feedback also underscore the importance of advancing rehabilitation technologies, such as the development of robotic devices and biofeedback systems, to support individualized and scalable interventions. Future research should also focus on the development of hands-free technologies to make the combined feedback intervention more practical and applicable to daily life.

## 5. Conclusions

Both groups demonstrated significant improvements in balance ability from pre- to post-intervention; however, no significant between-group differences were observed in BBS scores. Notably, the RAS-V group showed a significantly greater improvement in TUG times compared to the RG group. Regarding gait, the RAS-V group exhibited significant improvements in gait cadence, gait velocity, and performance on the 10 mWT after the intervention. Comparisons between the groups revealed statistically significant differences, further supporting the efficacy of the combined intervention. Stride length showed no significant changes within and between groups.

These results confirm that RAS gait training combined with vibrotactile feedback has a significant impact on improving balance and gait function, particularly gait velocity and stability, in individuals with stroke. The findings underscore the importance of integrating external feedback, such as vibrotactile cues, into walking training programs. This combined approach appears to be more effective in enhancing gait function compared to RAS alone.

## Figures and Tables

**Table 1:** 4-week intervention program of the RAS-V and RG groups.

Group	Intervention Contents	Session Time (Minute)
RAS-V	Conventional physical therapy (NDT)	30
	Warm up	3
	RAS + Vibrotactile feedback	25
	Cool down	2
RG	Conventional physical therapy (NDT)	30
	Warm up	3
	RAS	25
	Cool down	2

Abbreviations: RAS-V, Rhythmic auditory stimulation gait training combined with vibrotactile feedback; RG, Rhythmic auditory stimulation gait training; RAS, Rhythmic auditory stimulation.

**Table 2:** Clinical demographic characteristics of study patients (n = 22).

Variables	RAS-V Mean ± SD	RG Mean ± SD	x2/t	*p*
Sex, male/female	5/6	7/4	0.733	0.670
Age, years	70.27 ± 13.33	63 ± 11.77	1.356	0.190
Weight, kg	64.64 ± 7.85	57.73 ± 7.95	2.051	0.054
Height, cm	162.36 ± 10.99	163.45 ± 7.55	0.271	0.789
Lesion type, Inf/Hrr	9/2	7/4	0.917	0.635
Lesion side, Right/Left	5/6	8/3	1.692	0.387
MMSE-K, score	25.91 ± 1.81	26.36 ± 1.91	0.572	0.574

Abbreviations: RAS-V, Rhythmic auditory stimulation gait training combined with vibrotactile feedback; RG, Rhythmic auditory stimulation gait training; Inf, Infarction; Hrr, Hemorrhage; MMSE-K, Korean version of Mini-Mental Status Examination.

**Table 3:** Baseline characteristics of clinical dependent variables (n = 22).

	RAS-V Mean ± SD	RG Mean ± SD	t	*p*
BBS, score	22.82 ± 8.80	27.27 ± 9.64	1.132	0.271
TUG, s	30.70 ± 16.33	20.75 ± 6.72	1.869	0.076
Cadence, steps/min	78.23 ± 16.68	88.74 ± 15.84	1.516	0.145
Velocity, m/s	0.91 ± 0.34	1.19 ± 0.42	1.735	0.098
Stride length, cm	1.42 ± 0.33	1.61 ± 0.38	1.280	0.215
10 mWT, s	26.41 ± 13.52	19.41 ± 7.35	1.508	0.147

Abbreviations: RAS-V, Rhythmic auditory stimulation gait training combined with vibrotactile feedback; RG, Rhythmic auditory stimulation gait training; BBS, Berg Balance Scale; TUG, Timed Up and Go test; 10 mWT, 10 m Walk Test.

**Table 4:** The comparison of balance ability values between the RAS-V and RG groups.

		RAS-V Mean ± SD	RG Mean ± SD	t	*p*	d	95% CI
BBS, score	Pre	22.82 ± 8.80	27.27 ± 9.64				
	Post	27.82 ± 9.78	31.00 ± 9.18				
	Post-Pre	5.00 ± 3.26	3.73 ± 2.10	1.089	0.29	0.46 ^†^	(−0.39, 1.31)
	t	5.093	5.881				
	*p*	0.000	0.000				
TUG, s	Pre	30.70 ± 16.33	20.75 ± 6.72				
	Post	26.18 ± 13.52	19.70 ± 6.29				
	Post-Pre	4.53 ± 3.82	1.05 ± 0.92	2.931	0.013	1.25 ^‡^	(0.47, 2.04)
	t	3.929	3.778				
	*p*	0.003	0.004				

Abbreviations: RAS-V, Rhythmic auditory stimulation gait training combined with vibrotactile feedback; RG, Rhythmic auditory stimulation gait training; BBS, Berg Balance Scale; TUG, Timed Up and Go test; CI, Confidence Interval. ^†^ Moderate effect size, ^‡^ Strong effect size.

**Table 5:** The comparison of gait ability values between the RAS-V and RG groups.

		RAS-V Mean ± SD	RG Mean ± SD	t	*p*	d	95% CI
Cadence, steps/min	Pre	78.23 ± 16.68	88.74 ± 15.84				
	Post	91.61 ± 17.92	91.37 ± 17.76				
	Post-Pre	13.38 ± 7.53	2.63 ± 8.21	3.202	0.004	1.36 ^‡^	(0.57, 2.16)
	t	5.893	1.061				
	*p*	0.000	0.31				
Velocity, m/s	Pre	0.91 ± 0.34	1.19 ± 0.42				
	Post	1.10 ± 0.35	1.20 ± 0.47				
	Post-Pre	0.19 ± 0.22	0.01 ± 0.09	2.470	0.028	1.07 ^‡^	(0.31, 1.84)
	t	2.851	0.471				
	*p*	0.017	0.65				
Stride length, cm	Pre	1.42 ± 0.33	1.61 ± 0.38				
	Post	1.61 ± 0.38	1.66 ± 0.43				
	Post-Pre	0.19 ± 0.34	0.05 ± 0.10	1.374	0.19	0.56 ^†^	(−0.17, 1.29)
	t	1.894	1.549				
	*p*	0.09	0.15				
10 mWT, s	Pre	26.41 ± 13.52	19.41 ± 7.35				
	Post	21.30 ± 11.71	18.75 ± 7.51				
	Post-Pre	5.11 ± 4.92	0.65 ± 1.91	2.798	0.015	1.20 ^‡^	(0.42, 1.97)
	t	3.441	1.138				
	*p*	0.006	0.28				

Abbreviations: RAS-V, Rhythmic auditory stimulation gait training combined with vibrotactile feedback; RG, Rhythmic auditory stimulation gait training; 10 mWT, 10 m Walk Test; CI, Confidence Interval. ^†^ Moderate effect size, ^‡^ Strong effect size.

## Data Availability

All data generated or analyzed during this study are included in this published article.
